# Tiagabine treatment in kainic acid induced cerebellar lesion of dystonia rat model

**DOI:** 10.17179/excli2016-482

**Published:** 2016-11-17

**Authors:** Tsui-chin Wang, Sukonthar Ngampramuan, Naiphinich Kotchabhakdi

**Affiliations:** 1 Research Center for Neuroscience, Institute of Molecular Biosciences, Mahidol University, Salaya campus, Nakhon Pathom 73170, Thailand

**Keywords:** dystonia, cerebellum, kainic acid, tiagabine, telemetry recording

## Abstract

Dystonia is a neurological disorder characterized by excessive involuntary muscle contractions that lead to twisting movements. The exaggerated movements have been studied and have implicated basal ganglia as the point of origin. In more recent studies, the cerebellum has also been identified as the possible target of dystonia, in the search for alternative treatments. Tiagabine is a selective GABA transporter inhibitor, which blocks the reuptake and recycling of GABA. The study of GABAergic drugs as an alternative treatment for cerebellar induced dystonia has not been reported. In our study, tiagabine was i.p. injected into kainic acid induced, cerebellar dystonic adult rats, and the effects were compared with non-tiagabine injected and sham-operated groups. Beam walking apparatus, telemetric electromyography (EMG) recording, and histological verification were performed to confirm dystonic symptoms in the rats on post-surgery treatment. Involuntary dystonic spasm was observed with repetitive rigidity, and twisting movements in the rats were also confirmed by a high score on the dystonic scoring and a high amplitude on the EMG data. The rats with tiagabine treatment were scored based on motor amelioration assessed via beam walking. The result of this study suggests and confirms that low dose of kainic acid microinjection is sufficient to induce dystonia from the cerebellar vermis. In addition, from the results of the EMG recording and the behavioral assessment through beam walking, tiagabine is demonstrated as being effective in reducing dystonic spasm and may be a possible alternative therapeutic drug in the treatment of dystonia.

## Abbreviations in the Experiment

preS: pre-surgery, postS: post-surgery, sham: sham-operated group, KA: kainic acid/non-treated group, KA+TGB: kainic acid tiagabine treated group, TGB: tiagabine group, BW: beam walking, EMG: electromyography, DV: dorsal-ventral coordinates, i.p.: intraperitoneal injection.

## Introduction

Dystonia is a neurological disorder characterized by involuntary and prolonged muscle contractions that leads to twisting movements or abnormal posturing (Neychev et al., 2008[22]). The dystonic symptoms are stereotyped and repetitive as a result of the agonist and antagonist muscle co-contraction that induce affected body parts to over twist (Tanabe et al., 2009[31]). When the abnormal movement continues for a period of time, unaffected body parts are forced into stiff abnormal posture, relatively (LeDoux and Brady, 2003[15]; LeDoux, 2011[14]). Dystonia can also be task-specific, where patients are normal until they begin to perform certain tasks, such as the writing or playing a musical instrument resulting in writer's cramp or musician's cramp (Quartarone et al., 2006[27]). Dystonia is regionally specific such as oromandibular dystonia that interferes with speech, blepharospasm that leads to the involuntary closing of the eyelids (Breakefield et al., 2008[6]), and cervical dystonia that causes uncontrolled neck movement (van de Warrenburg et al., 2007[32]). 

It was believed that dystonia is an inherited disorder and is age-onset dependent, considered as primary dystonia, since no neurological lesions have been found in association with the disorder (Bhidayasiri, 2006[5]). Some cases of dystonia may accompany other neurological disorders such as brain injury or Parkinson's disease; they are categorized as secondary dystonia (Breakefield et al., 2008[6]). Although dystonia has recently been successfully treated by deep brain stimulation (DBS) in specific regions of basal ganglia (Krauss, 2002[13]; Ostrem and Starr, 2008[24]), the origin and neural mechanisms of dystonia involving alternative brain circuitry have not been explained. 

Many GABA transporter subtypes have been cloned as the target for treatment of convulsions and seizures (Madsen et al., 2007[18]; Roth and Draguhn, 2012[28]). The GABA molecules that encounter uptake by transporters (GAT1, GAT3) at the neuronal terminals can be recycled in the process of GABA transmission (Madsen et al., 2010[20]). GAT1 has been found in the cerebellar layers, with high concentration in the Purkinje cell layer and molecular layer (Itouji et al., 1996[10]). Tiagabine (TGB) is a GAT1 inhibitor, which enhances GABA effects (Madsen et al., 2011[19]) and controls CNS excitability (Schousboe et al., 2004[29]). As a lipophilic derivative of nipecotic acid, TGB crosses the blood-brain barrier to modulate intra-synaptic GABA reuptake (Azar and Abou-Khalil, 2010[4]), but does not increase GABA concentration in the cerebrospinal fluid (CSF) (Angehagen et al., 2003[3]). Tiagabine has been investigated as an anti-seizure treatment in the GABAergic system. In human clinical studies, tiagabine was well tolerated and did not affect GABA plasma concentration (Azar and Abou-Khalil, 2010[4]; Crawford et al., 2001[9]). Although some patients experienced side effects including dizziness, there were significant reductions in partial, complex partial and generalized tonic-clonic seizures (Crawford et al., 2001[9]), as well as transient acute dystonic movements (Wolanczyk and Grabowska-Grzyb, 2001[33]). TGB also reduces convulsions and ataxia (Madsen et al., 2011[19]), and alleviates muscle spasticity induced by transient spinal ischemia (Kakinohana et al., 2012[12]).

Some studies have investigated involvement of cerebellar lesion in dystonic rigidity; however, research on possible pharmacological treatment for dystonia remains limited. The aim of the present study was to investigate tiagabine as a possible treatment for kainic acid induced dystonia in the cerebellum. 

## Materials and Methods

### Animals

**Figure 1:**
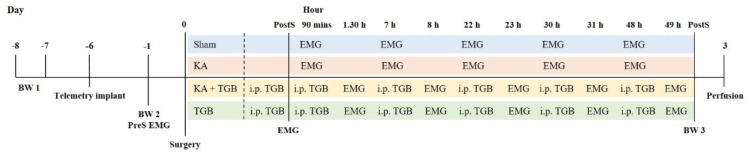
Experimental outline The experimental outline is divided into 2 parts, pre-surgery and post-surgery, where the day of the stereotaxic surgery is regarded as day 0. About one week before the stereotaxic surgery (-8 and -7 day), the rats were trained for beam walking for the 1^st^ time (BW1). The next day, the rats received a telemetry implant (-6 day) and were allowed to rest for a few days before their 2^nd^ training session for beam walking and the pre-surgery EMG recording (-1 day, BW2, preS EMG). On the day of the surgery, they were divided into 4 groups: the sham group with saline injection, the KA group with kainic acid injection, the KA+TGB group with kainic acid injection and tiagabine i.p. injection, and the TGB group with tiagabine i.p. injection only. All rats were recorded for post-surgery EMG (postS EMG) immediately after they recovered from surgery. Another recording was conducted at 90 minutes, 7^th^, 22^nd^, 30^th^, and 48^th^ hours after surgery for the sham and the KA group, while the KA+TGB and the TGB groups received another tiagabine i.p. injection at 90 minutes after surgery and recorded for EMG one hour later (1.30 h), and continued at the 7^th^, 22^nd^, 30^th^, and 48^th^ hours post-surgery. At the 49^th^ hour post-surgery, all rats were tested in the 3^rd^ beam walking session and then sacrificed 3 days after the surgery. BW1 and BW2 are considered as pre-surgery BW testing. BW3 is considered as post-surgery testing. BW, beam walking; preS, pre-surgery; EMG, electromyography; sham, sham-operated; KA, kainic acid; KA+ TGB, kainic acid with tiagabine treatment; TGB, tiagabine only; i.p., intraperitoneal; h, hour; postS, post-surgery

Adult male Sprague Dawley rats (11 weeks old, weighing 250-270 g) were obtained from the National Laboratory Animal Center, Thailand. The rats were kept individually in the stainless steel, solid bottom, opened top cages with a 12/12 light-dark cycle, and with rat chow and water *ad libitum*. Animals were weighted and fed every day at the same time. All experimental procedures were approved by the Animal Care and Use Committee of the Mahidol University, Thailand (MB-ACUC 2012/02). The experimental procedure of this study is summarized in Figure 1(Fig. 1).

### Concentration and coordinate assessment 

**Figure 2:**
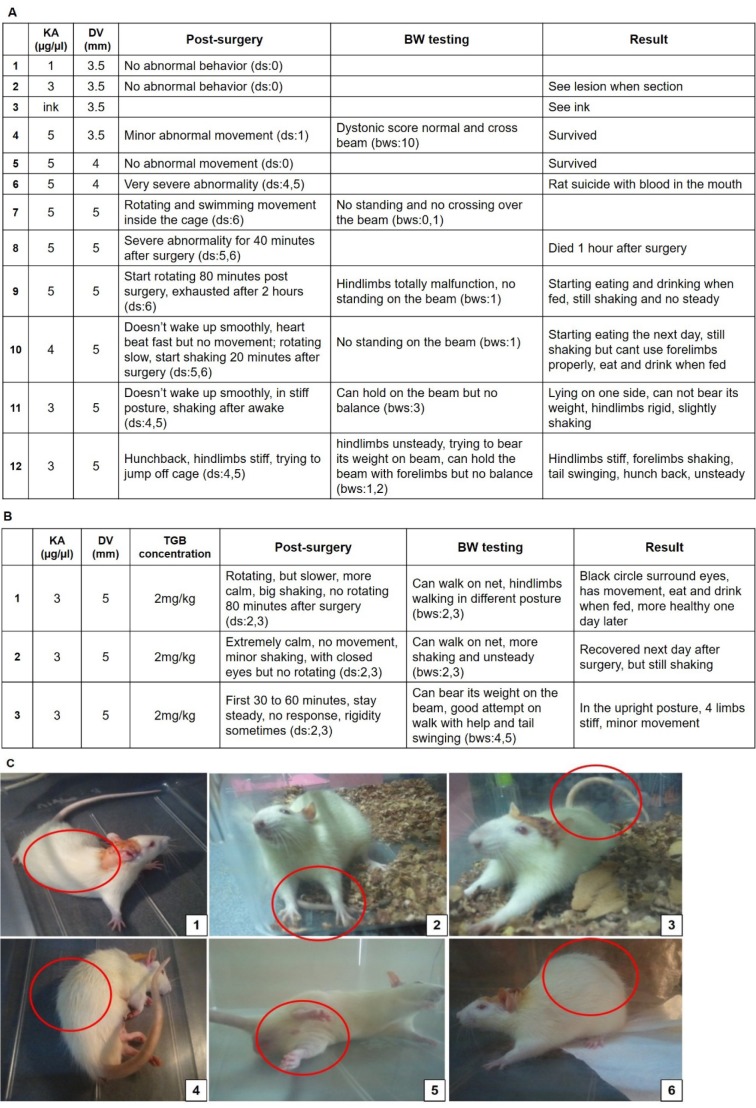
Concentration-coordinates assessment (A) Kainic acid concentrations were matched with distinct dorsal-ventral coordinates of the rat brain to determine the rat behavior and response after the stereotaxic surgery. In the first group of testing (trial 1 to 4), kainic acid at 3 and 5 µg/µl was injected to a depth of 3.5 mm, which revealed normal movements, giving a dystonia score of 0 and a beam walking score of 10. With further injection to the dorsal location of the brain, where DV was at 4 or 5 mm (trial 5 to 9), the rats showed rotating and over-excited epileptic-like seizures, with a dystonia score of 6 and a beam walking score of 0 and 1. Therefore, the kainic acid concentration was reduced to 4 µg/µl and then re-tested with 3 µg/µl (trial 10 to 12) the rats showed stiffness, with the dystonia scores of 4 and 5, and the capacity to hold on the beam (beam walking scores of 3). (B) With the confirmation of kainic acid concentration at 3 µg/µl and DV 5 mm, tiagabine concentration was set at with 2 mg/kg. The figure shows that the rats are steadier in the upright position, with less rigidity and a capacity to walk on the beam after one dose of tiagabine. The dystonia score was 2 to 3 and the beam walking score was 4 to 5. (C) The rats were observed with trunk twisting (1, 5) and hunchback (1, 4, 6), head and tail unsteadily swinging (3, 4), limb stiffness (2, 3, 6) and with dystonic seizure after awakening from the stereotaxic surgery. KA, kainic acid; DV, dorsal-ventral coordinates; BW, beam walking; TGB, tiagabine; i.p., intraperitoneal; ds, dystonia scores from Table 1; bws, beam walking scores from Table 2.

Kainic acid and tiagabine concentration assessment was performed before the onset of the experiment in order to select kainic acid concentration; microinjection coordinates in the cerebellum, the animal tolerance to the drug and the treatment outcome. In the concentration comparison testing, kainic acid concentration and dorsal-ventral (DV) coordinates were observed as part of the rat's response after they awaked from anesthesia. In addition, the tolerance to drugs the next few days after the kainic acid microinjection was recorded (Figure 2A(Fig. 2)). Tiagabine concentration was also investigated in the concentration comparison testing (Figure 2B(Fig. 2)). The aim of the second concentration comparison testing was to consider different concentrations of tiagabine i.p. injection and to observe the drug efficiency 90 minutes after the first injection. Dystonia behavior phenotype scoring (Table 1(Tab. 1)) and beam walking scoring (Table 2(Tab. 2)) were used. Abnormal posture of dystonic rats were also observed as in Figure 2C(Fig. 2). 

### Telemetric transmitter implantation

Telemetric transmitter (TA11CTA-F40, Data Sciences International, St. Paul, MN, USA) was prepared before the surgery. Insulation tubing of the transmitter electrode was removed from the distal part of the lead and a loop was formed (Cesarovic et al., 2011[8]). The transmitter was then sterilized with Septichlor. Rats were anesthetized with Isoflurane, 3.5 % in 100 % oxygen). Following midline abdominal incision, the transmitters were implanted intraperitoneally. The electrodes were tunneled subcutaneously and fixed to the gastrocnemius and tibialis anterior muscles using silk sutures (Cesarovic et al., 2011[8]). Carproten (Laboratonos, Pfizer, rail) 2.5 mg/kg and Enrofloxacin (General Drugs House Co., LTD, BKK, Thailand) were injected to reduce pain and prevent inflammation every 12 hours, for 3 days.

### Stereotaxic surgery and tiagabine injection

After the telemetry implantation, the rats were randomly divided into 4 groups. There were the sham-operated group (sham), the kainic acid group (KA), the kainic acid with tiagabine treatment group (KA+TGB), and the tiagabine group (TGB). The sham, KA, and KA+TGB groups received stereotaxic surgery. On the day of the surgery, all the rats were weighed and abstained from food and water from the night before. Isoflurane (3.5 % in 100 % oxygen) was used in this surgery as anesthesia. After the rats were fully anesthetized, a midline incision was made in between the rat's' ears. The skull was drilled at the midline above the cerebellum and 1 µl of kainic acid (Sigma-Aldrich, 3 µg/µl) was microinjected (AP from interaural line: -2.4 mm, DV: -5 mm), for both the KA and the KA+TGB groups (Paxinos and Watson, 1982[25]). Tiagabine (Sigma-Aldrich, 2 mg/kg) was injected i.p. 5 minutes after the kainic acid microinjection. The sham group was microinjected with normal saline in placed of kainic acid. The tiagabine group received tiagabine injection at the same time, with the same concentration instead of receiving stereotaxic surgery. The concentration of kainic acid (3 µg/µl) and tiagabine (2 mg/kg), as well as dorsal ventral coordinates (DV: -5 mm) had been confirmed with preliminary experimentation (Figure 2(Fig. 2)). Once the rats were awakened from the anesthesia, as mentioned previously in the section of telemetry implantation, 2.5 mg/kg each of Carproten and Enrofloxacin were injected intraperitoneally and subcutaneously to reduce pain and prevent inflammation every 12 hours, for 3 days. 

### Dystonic movement observation and EMG recording 

The rats were moved from the surgery room to record their EMG amplitude and movements immediately after they recovered from anesthesia. Involuntary twisting and rigidity were scored using the dystonic phenotype table (modified from Pizoli et al., 2002[26]; Jinnah et al., 2005[11]; and Alvarez-Fischer et al., 2012[1], Table 1). 

A research assistant observed and measured the scores according to the assigned Table 1(Tab. 1) to avoid possible bias. A score of 1 represents a behavior pattern that reflects normal motor behavior, with normal posture. A score of 2-4 indicates dystonia with increasing severity. If limited walking and movement were shown, the rat was disturbed by touch. If there was no walking or moving at all and the rat couldn't stay in an upright position, it was lifted by tail and placed down again to encourage movement, which often preceded the dystonia (Pizoli et al., 2002[26]). The rat was observed lying still with continuous prolonged rigidity, giving a score of 5. If one showed twisting movement, it would be given score of 6. According to previous studies, dystonic rats scored about 4, 5, and 6. Normal rats scored 0 or 1. Therefore, the present study would also consider scores 4, 5, and 6 for dystonic rats, and scores 0 and 1 for sham-operated rats. 

At the same time, for dystonic testing and scoring, the rats were recorded for their 1^st^ post-surgery EMG amplitude. Each EMG recording was 30 minutes and the rats were taken for recordings at 90 minutes, 7 hours, 22 hours, 30 hours and 48 hours after surgery (Figure 1(Fig. 1)). Each EMG recording was video-taped for further comparison and analysis. 

### Beam walking assessment

The aim of the beam walking apparatus was to measure the foot slip and the latency to transverse the beam for motor coordination and balance (Carter et al., 2001[7]). The size of the beam used in this parameter was 50 mm x 150 cm. The starting point was marked at the 0 cm line and the endpoint at the 110 cm. Every 10 cm was labelled with a permanent ink to record the distance. A net was attached under the beam to prevent injuries from falling and escaping. Two 40-watt lamps were set above and at the starting point as an adverse stimulus and a goal box was set by the end point to encourage forward movement (Carter et al., 2001[7]). A video recording camera was fixed in front of the starting point (Stanley et al., 2005[30]). Each rat was trained with 3 consecutive trials in 3 days (Luong et al., 2011[17]), including 2 days before the telemetry implant (BW1, Figure 1(Fig. 1)) and 1 day after the implant (BW2, Figure 1(Fig. 1)), as pre-surgery session. For the post-surgery session, the rats were given 60 seconds on the bridge per trial and the one that fell would be returned to the spot of the fall (Stanley et al., 2005[30]). For the ones that failed to pass the bridge within 60 seconds, the distance travelled was recorded. The time and distance of travel were recorded for each trial, before and after the surgery. The rat behavior on the beam was observed and scored for comparison between groups using the beam walking testing (Table 2(Tab. 2), modified from Anand et al., 2011[2]). Weight bearing and walking ability were used to determine the scores. Animals with no weight bearing were given 0 to 1 points, ones with minor or mild weight bearing were given 2 to 3, and ones with good weight bearing were given 4-5 points. The ones able to walk within 60 seconds with minor foot slips were given 6 to 10 points (Anand et al., 2011[2]). The immobilized ones on the beam were given less points and the ones that passed the beam were given higher points. The scores given are different from the dystonic scores. The scores were assigned by a research assistant to avoid possible bias. 

### Animal sacrifice and brain collection

After the perfusion procedures, brains were collected and post fixed with 4 % formaldehyde for one week and then changed to 30 % sucrose solution. The brain was sectioned in paraffin sections with microtome. The sections were stained with hematoxylin and eosin staining (H&E staining), and taken for whole brain imaging for needle tracing. A stereomicroscope was used to take a whole brain image, with an objective lens of 1.25X.

### EMG data acquisition and analysis

The analogue EMG was digitized at 1 KHz and acquired using PowerLab A/D converter and Chart 5.4 software (AD Instruments, Australia) (Ngampramuan et al., 2008[23]). The results measured the peak amplitude of muscle contraction after kainic acid microinjection and/or tiagabine i.p. injection for 30 minutes. EMG variability analysis in the amplitude domain was performed using Chart 5.4 software. 

### Data collection and analysis 

One-way ANOVA with Turkey's multiple comparison tests was used to calculate the statistical differences for dystonic behavioral phenotype score, EMG amplitude comparison, and beam walking observational scoring test. Sham-operated group and experimental groups were compared to reach statistical difference when p value is less than 0.05. Analysis was performed using Prism (GraphPad) software.

## Results

### Determination of drug concentration and coordinates for the microinjection

Using concentration-coordinates table (Figure 2A(Fig. 2)), the animal status was scored after awakening from surgery. It indicated the immediate tolerance of a rat towards the drug. When tested with various concentrations of kainate (1, 3, and 5 µg/µl) to DV 3.5 mm, only the concentration of 5 µg/µl resulted in the expression of minor abnormal movements, with a dystonia score of 1 (ds:1), obtained from Table 1(Tab. 1). However, when running on the beam, there was no loss of balance or the capability of weight bearing, and the rats that could walk across the beam scored a 10 for the beam walking observation (bws:10, obtained from Table 2(Tab. 2)). When microinjection was made at the depth of 4 to 5 mm, the rats could not tolerate the drug toxicity, expressing their intolerance by over-twisting and running in circles inside the cage (ds: 6), which resulted in bleeding from their mouth. The rats became over exhausted and paralyzed the day after the surgery (ds: 6), some even died before the end point of the experiment. As DV was confirmed to cause the lesion, we needed to keep the animals alive by reducing the kainic acid concentration. After a few trials of 3 µg/µl vs. the 5 mm testing, we agreed on the degree of stiffness and rigidity (ds: 4, 5) as well as the ability to hold on the beam (bws: 3), which was considered as the optimal concentration and coordinates (Figure 2A(Fig. 2)). In this study, 2 mg/kg tiagabine concentration was i.p. injected post-kainic acid microinjection (Figure 2B(Fig. 2)). This concentration was also examined with kainic acid concentration and DV coordinate obtained from Figure 2A(Fig. 2). The animals showed more steadiness and calmness 80 to 90 minutes post-surgery (ds: 2, 3) and were able to walk on the beam (bws: 4, 5). 

**Figure 3:**
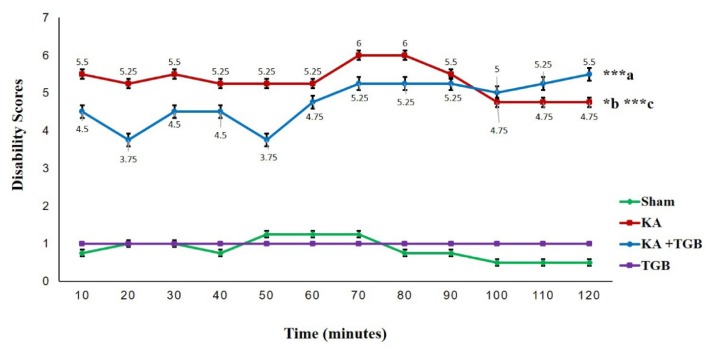
Dystonic behavioral phenotype score This Figure shows the behavioral observation within 2 hours duration post-surgery for 4 groups: sham, KA, KA+TGB, and TGB. The scores were obtained from Table 1. The KA group shows a gradually higher score compared to sham (***c) and KA+TGB (*b). KA+TGB also had higher scores compared with sham (***a). The TGB group did not go through the stereotaxic surgery, so they remained steady. Significantly different value: ***a/c, p<0.001; *b, p<0.05; Sham, sham-operated; KA, kainic acid; KA+ TGB, kainic acid with tiagabine treatment; TGB, tiagabine only group.

**Figure 4:**
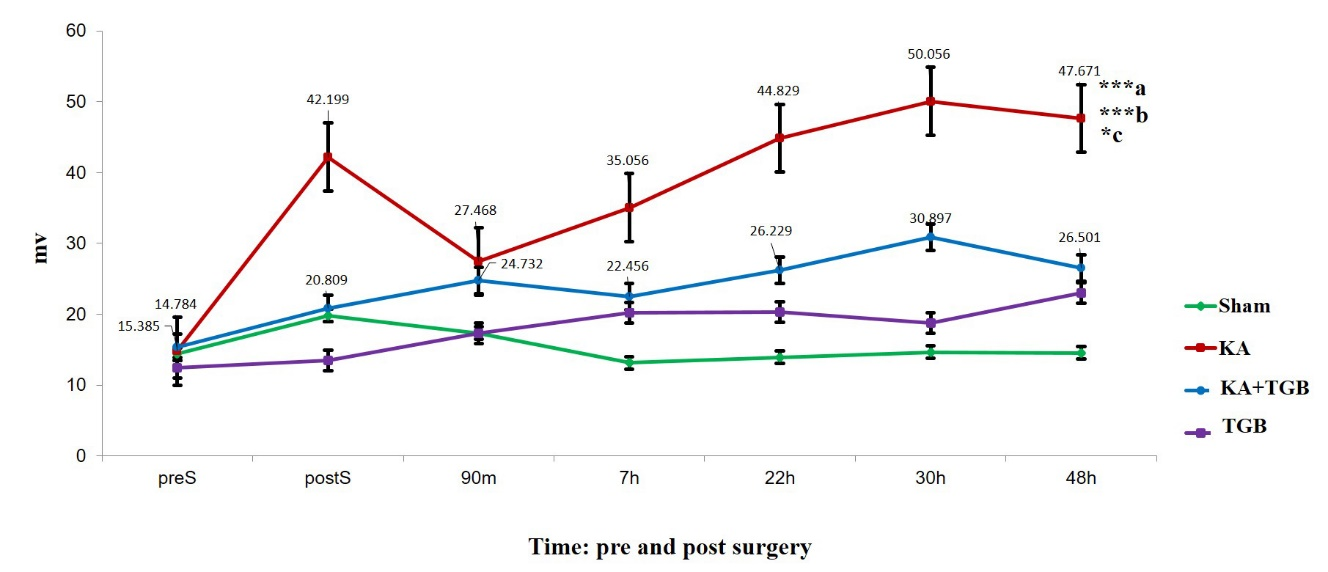
EMG amplitude comparison This Figure compares the average EMG amplitude between the 4 groups; sham, KA, KA+TGB and TGB. The kainic acid group had significant gradual bursting in the EMG with more severity in dystonic rigidity and muscle spasm compared with sham (***a), with KA+TGB (*c,); with TGB group (***b). The numbers above, or beside the plotted points, show the mean ± SEM (standard error of mean) for only the treatment group (KA+TGB) and the non-treatment group (KA). Significantly different value: ***a/b, p<0.001, *c, p<0.05. preS, pre-surgery; postS, post-surgery; m, minute; h, hour; Sham, sham-operated; KA, kainic acid; KA+ TGB, kainic acid with tiagabine treatment. TGB, tiagabine only.

**Figure 5:**
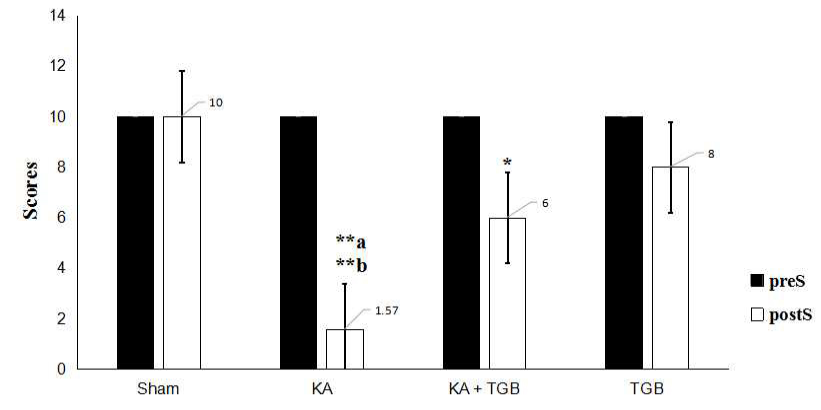
Beam walking behavioral observation The scores were obtained from Table 2; when the rats were running or holding onto the beam, with the objective of quantifying the rats' motor behavior and balance. The KA group, compared to the sham group (**a) and the TGB group (**b), expressed most severe dystonic seizures with difficulty staying on the beam or starting walking. The average score was 1.57 for the KA+TGB group compared with to KA group (*p<0.5). The average score of 6 was generally given to the rats for walking through the beam within 60 seconds with fewer falls and significantly different value: **a/b p< 0.01, *p<0.5. preS, pre-surgery; postS, post-surgery; Sham, sham-operated; KA, kainic acid; KA+TGB, kainic acid with tiagabine treatment. TGB, tiagabine only.

**Figure 6:**
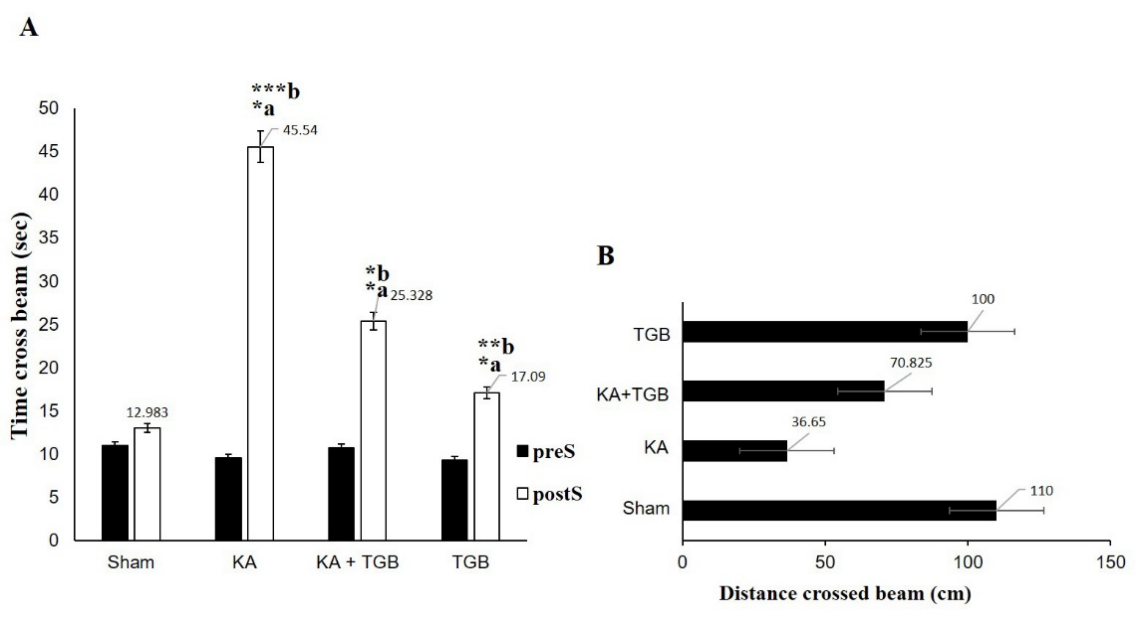
Time and distance crossed the bridge These two Figures describe time and distance used to cross the beam in the 4 groups: sham, KA, KA+TGB, and TGB groups. (A) In comparing the pre- and post-surgery data for each group, all the rats used significantly longer time after surgery (KA: *a; KA+TGB: *a; TGB: *a). After the surgery, KA also spent significantly longer time (45 seconds in average) than sham (***b), KA+TGB (*b), and TGB (**b). (B) Distance in crossing the beam. Although the KA group spent the longest time on the beam, this group only travelled 36 cm on average. KA+TGB, the treatment group, travelled 70 cm on average. Significantly different value: *a/b, p<0.05, **b, p<0.005, ***b, p<0.0005. preS, pre-surgery; postS, post-surgery; Sham, sham-operated; KA, kainic acid; KA+ TGB, kainic acid with tiagabine treatment; TGB, tiagabine only.

The abnormal behavior was expressed after the rats awakened from the surgery. Limb stiffness and rigidity, and twisting trunk could be observed (Figure 2C(Fig. 2), 1 and 5). Some rats expressed a hunchback posture, lying on one side with limbs freely swinging (Figure 2C(Fig. 2), 4). Some could stand on their feet, but if the tail was lifting up, their limbs would attach against their trunk, indicating no support from the lower part of the body and hind limbs (Figure 2C(Fig. 2), 6). The day after the surgery, although the rats started eating and drinking, they still experienced limb stiffness with their tails swinging in various directions from time to time (Figure 2C(Fig. 2), 2 and 3).

### Test for disability scoring for dystonic severity

After the animals awakened from anesthesia, they had post-surgery EMG recordings. The abnormality and rigidity of the dystonic seizure was videotaped for a later dystonic scoring by a third person. The severity of the dystonic seizure within the period of 120 minutes post-surgery was given scores according to Table 1(Tab. 1).

According to the graph, the KA group maintained a score between 5 and 6; in contrast, there was a score variation in the KA+TGB group, which finally stabilized at 60 minutes post-surgery (Figure 3(Fig. 3)). There was a little shift in scores from 100 minutes until 120 minutes post-surgery, where the KA group (scores 4.75, 4.75, 4.75, in average, p<0.05) scored slightly less compared with the KA+TGB group (scores 5, 5.25, 5.5, in average), which correlates with data on the EMG observational scoring table at the 90 minutes (Figure 4(Fig. 4)). These phenomena suggest that tiagabine treatment meets the peak efficiency, and the acute injection of kainic acid functioned as the stabilizing effect of the dystonic seizures. Please refer to supplemental data (S1-Table 1) for complete scores of individual rats.

### Intra-muscular EMG recording

After the rats received the EMG telemetry implantation, there were seven EMG recordings (preS, postS, 90 minutes, 7 hours, 22 hours, 30 hours and 48 hours) (Figure 1(Fig. 1)). Each EMG recording was 30 minutes, except for the pre-surgery, 15 minutes.

For post-surgery recordings, the rats were recorded only after awakening from anesthesia. The KA group showed most exaggerated muscle contractions compared to the sham, KA+TGB and TGB groups (Figure 4(Fig. 4)). There was a high bursting frequency post-surgery (42.199±11.122), although muscle was relaxed with less amplitude at 90 minutes post-surgery (27.468±11.091), the amplitude was stabilized at the 7^th^ hour post-surgery (35.056±8.924) and gradually increased until the 30^th^ hour post-surgery (50.056±5.862). Please refer to supplemental data (S2-Table 1) for the complete standard deviation value.

Kainic acid-injected rats expressed highest amplitude in muscle contraction, which was significantly different when compared to sham-operated animals (p< 0.001). The KA group also expressed significantly higher amplitude compared with the KA+TGB group (p< 0.05). In addition, the KA group also showed significant difference compared to the TGB group (p< 0.001). In comparison with the KA group, the KA+TGB group showed an increase in amplitude gradually from postS (20.809±4.058) to 90 minutes (24.732±5.341), and also stabilized at 7 hours (22.456±3.885). From the graph, the KA and the KA+TGB groups composed a correlated slope from 7 hours to 48 hours (Figure 4(Fig. 4)). Please refer to supplemental data (S2-Table 1) for the complete standard deviation value.

### Beam walking analysis 

All rats were trained for beam walking for 2 sessions before the kainic acid microinjection (BW1 and BW2, Figure 1(Fig. 1)). After full recovery from the kainic acid microinjection, the rats were required to walk on the beam again (BW3, Figure 1(Fig. 1)). The purpose of the third attempt was to record the efficacy of the tiagabine treatment in that case of kainic acid excitotoxicity in the cerebellar cortex, as well as to make a comparison with the non-treated and pre-surgery groups. In this behavioral apparatus, we quantified not only the locomotion, but also the observation scores on how the animals responded to the beam walking after the surgery, with/without tiagabine treatment (Table 2(Tab. 2)). The scoring was given by a third person to avoid possible bias. 

As illustrated in Figure 5(Fig. 5), the post-surgery response reached a statistical difference compared to the pre-surgery condition. The kainic acid group had difficulty in weight bearing and was very sensitive when touching the beam (scored 1.57 in average). Post-surgery KA expressed a significantly different reaction towards the beam when compared with the post-surgery sham group (scored 10 in average, p<0.01), KA+TGB (scored 6 in average, p<0.5), and TGB (scored 8 in average, p<0.01) groups. KA+TGB rats had limited weight bearing, but could hold onto the beam with their limbs and crawl across the beam with some help (scored 6 in average), although it took longer time (Figure 6(Fig. 6)). TGB had no significantly different motion when touching the beam (scored 8 in average). They had good weight bearing and good crossing time, except for a few foot slips. Please refer to supplemental data (S3-Table 1) for the complete scores of individual rats.

Each rat was compared before and after the surgery to observe their behavioral changes, especially with the tiagabine treatment. In Figure 6A(Fig. 6), KA group had taken a significantly longer duration in holding on the beam (45 seconds in average), when compared with the sham (12 seconds in average, p<0.0005) and the TGB groups (17 seconds in average, p<0.005), which also spent more than 50 % of the time crossing the beam when compared with KA+TGB. Despite spending longer time on the beam walking task, rats in the KA group failed to pass the 110 cm beam completely (average 36 cm, Figure 6B(Fig. 6)). For the treatment group, although the rats did not completely traverse the beam either, they managed to spend significantly less time on the beam (25 seconds in average, p<0.05) and completed the task despite foot slips and the fails, with the distance travelled of 100 cm.

### Histological imaging

**Figure 7:**
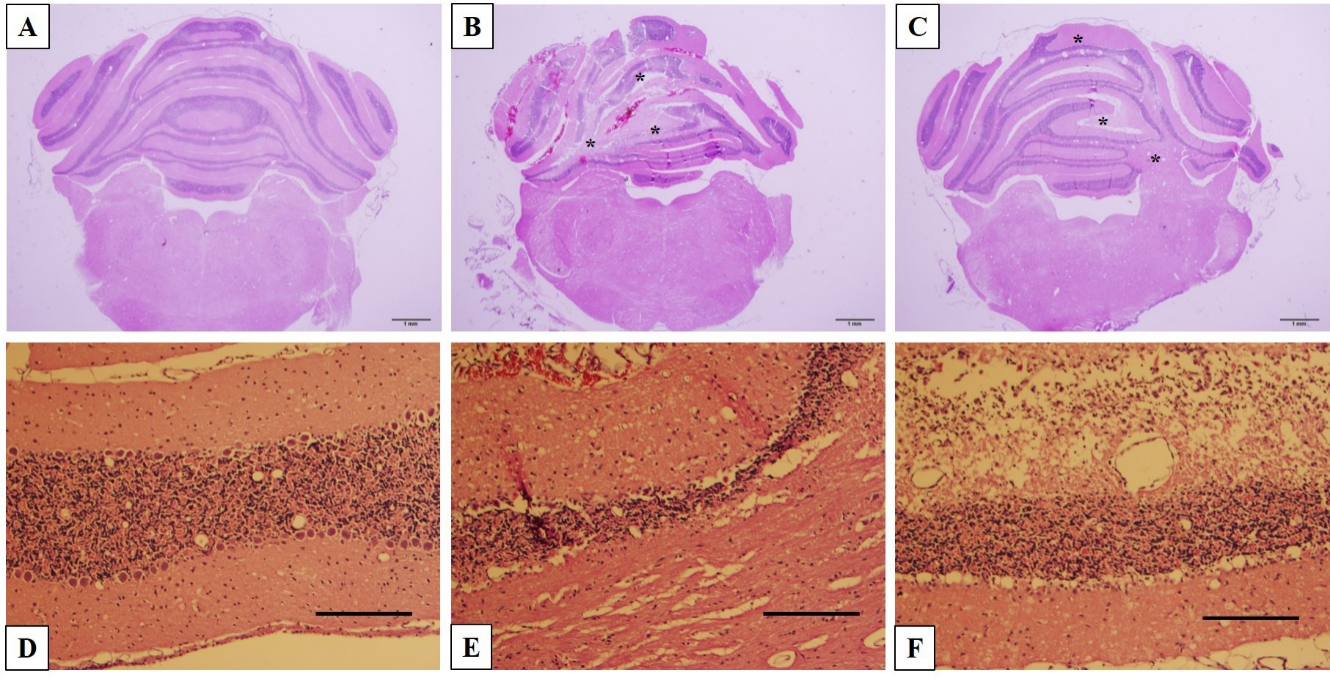
Histological images These Figures indicate cell loss and molecular layer fragmentation compared to the sham-operated group. The sham group shows dedicated cell layers of the cerebellum (A, D). There is needle tract lesion and cell loss in the KA group (B*) and layer disconnection in the KA+TGB group (C*). With higher magnification, KA shows thinner layers (E), but KA+TGB shows fibrous tissue and space enlargement (F) between cerebellar layers. A, D: sham group, B, E: KA group, C, F: KA+TGB group. Whole brain section 1.25X scale 1 mm; 10X magnification scale 200 µm.

All brains were collected, sectioned and stained with H&E to observe the morphology of cerebellar tissue and cells, especially the lesion parts. For the KA and KA+TGB groups, there were obvious needle traces, fragmentation, cell loss and fibrous tissue development (Figure 7(Fig. 7)). When compared with the distinct cerebellar layers in the sham group, cell loss and fragmentation indicated layers of atrophy and enlargement of the intra-layer space rather than complete layers (Figure 7B, C(Fig. 7)). When observed at higher magnification (10X), the KA group showed thinner layers, indicating cell loss, which is in contrast with the KA+TGB sections, where some regions of the cerebellar layer showed fibrosis and fragmentation (Figure 7E, F(Fig. 7)). Despite different degrees of tissue atrophy, there was significant degeneration in the cellular morphology of the KA+TGB group and the non-treated KA group. 

### Limitation of the study

Further molecular analysis or quantitative cell number estimation could be performed if the improvement of motor function resulted from tiagabine treatment and/or cellular plasticity and adaptation. Although preliminary study was performed to determine the suitable kainic acid concentration, electroencephalography (EEG) may also have been required to determine whether the dystonic seizure we induced by kainic acid injection is indeed dystonia rather than some other cerebellar syndromes, such as epilepsy and ataxia. Pizoli's study with kainic acid-induced cerebellar dystonia suggests that this kind of stiffness and rigidity can be considered as dystonic symptoms after EEG measurement, which was compared with the effects of other glutamate drugs in their study (Pizoli et al. 2002[26]).

## Discussion

The present study focused on kainic acid-induced dystonia in the cerebellum. Four methods were used in this study, including the use of a dystonic phenotype table, EMG recording from the hind limb muscles, behavior-related task, and the histological sectioning and staining of the cerebellar samples. Results obtained with this complex approach allowed us to conclude that symptoms observed in our rats following kainic-induced cerebellar lesions could be classified as dystonia.

Kainic acid microinjection induced the lesion only at the area of interest, while keeping the rest of the brain and peripheral tissues intact (Neychev et al., 2008[22]). Implanting the telemetry electrodes at the agonist and antagonist muscles measured the direct response of muscle activity that correlated with animal behavior (Cesarovic et al., 2011[8]) when the dystonic spasm occurred. 

Cerebellum is mainly in charge of balance and equilibrium (Manto, 2006[21]), and walking on the beam is one of the optimal behavioral methods to examine motor imbalance (Carter et al., 2001[7]).

After 4 assessments of the tiagabine-treated group (KA+TGB) and the non-treated (KA) group, there was a progressive amelioration in the beam walking locomotion (Figures 5(Fig. 5) and 6(Fig. 6)), in time to complete the task, the covered distance on the beam. EMG data also showed significantly different amplitudes recorded from TGB-treated and non-treated groups (Figure 4(Fig. 4)). There was partial cerebellar layer degeneration in the serial sections, compared to the sham-operated group (Figure 7(Fig. 7)). With the motor improvement in beam walking, the distinct but minor cellular plasticity in the cerebellar layer might be the reason for the rats walking smoothly with perfect balance on the beam bridge again. The results of this study correspond with and confirm our hypothesis. Moreover, tiagabine was also i.p. injected into the lesion rats. The drug might have additionally been diffused to other GABAergic neurons and interfered with their transmission. We have no clear knowledge as to whether the motor improvement is from the lesion area, from the intact regions of the cerebellum or from the other brain regions involved with motor activity. In addition, there was an overlap between the KA group and the KA+TGB group at 100 minutes post-kainic acid injection. Although this result correlates with the EMG result at 90 minutes, the result was different from previous studies (Pizoli et al., 2002[26]), where rats displayed little locomotor activity after 120 minutes of observation. Perhaps longer time might be needed for the dystonic rats. 

Efficacy of tiagabine post-lesion treatment was also demonstrated in this study. Unlike pre-lesion treatment (Lin et al., 2013[16]), we applied the drug only when we had confirmed the degree of dystonic stiffness and rigidity in the preliminary study (Figure 2(Fig. 2)). In other words, it's rational to apply the treatment after dystonia diagnosis.

## Conclusion

Overall, dystonia is not just a basal ganglia disorder. It is associated with the dysfunction of the motor circuit of the cerebello-thalamo-cortical tract. The cerebellar lesions determine the symptoms of dystonia. In this study, we demonstrated that the cerebellar dysfunction might lead to dystonia. Tiagabine, GABA-uptake inhibitor, might be a possible therapeutic treatment for cerebellar dystonia. The development of therapeutic effects may need more investigation. However, our study confirms that the animal model of dystonia, evoked by a small dose of kainic acid, is sufficient to induce cerebellar dystonia, which corresponds with previous studies. 

## Notes

Sukonthar Ngampramuan and Naiphinich Kotchabhakdi (Research Center for Neuroscience, Institute of Molecular Biosciences, Mahidol University, Salaya campus, Nakhon Pathom 73170, Thailand; Tel: +66-81-483-6066, Email: naiphinich@gmail.com) contributed equally as corresponding authors.

## Acknowledgements

The authors would like to thank Dr. Rapeepun Vanichviriyakit from the Faculty of Science, Mahidol University, for helping with immune-staining and paraffin sectioning and Ms. Kanda Puttapongpurk, for her assistance in animal surgery and score giving related to behavioral observations. The authors also much appreciate Associate Professor Eugene Nalivaiko, Dr. Sujira Mukda and Miss Amornrat Kampee's help, who have been responsible for manuscript editing and grammar correction. 

## Disclosure

There is no conflict of interest between the authors of this paper.

## Supplementary Material

Supplementary information

## Figures and Tables

**Table 1:**
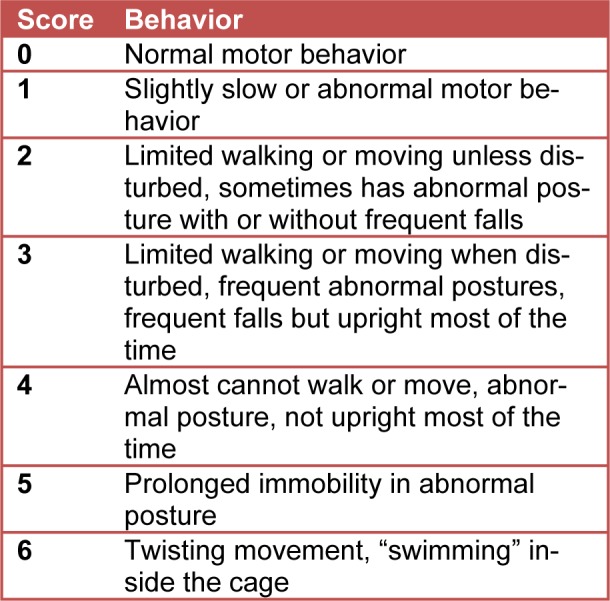
Dystonia behavioral phenotype The behavior table describes the posture, walking ability and steadiness of the rats after kainic acid surgery. Dystonia of score 0 is normal while 6 represents a serious dystonic condition with seizures and involuntary rigidity. Normal rats scored 0 to 1 and dystonic rats scored 4-6.

**Table 2:**
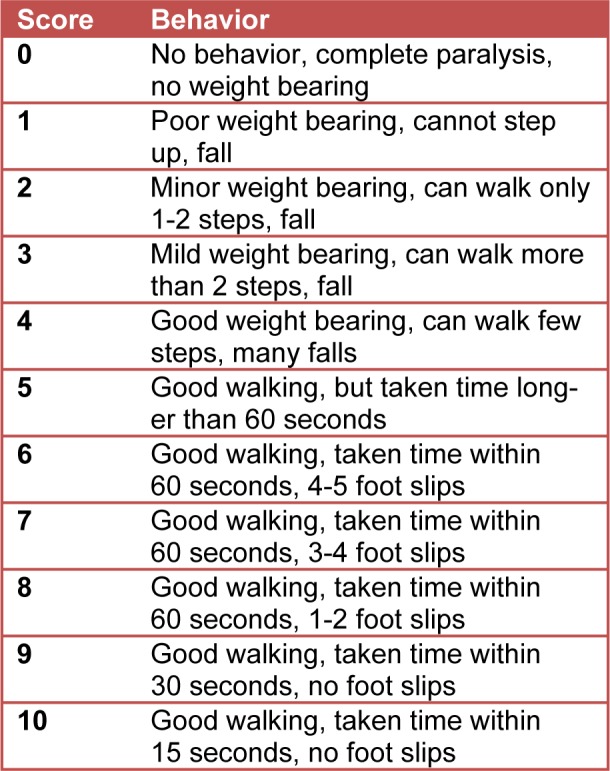
Beam walking observation This table describes the motor behavior and assigns the scores to individual rats while they were moving along the beam walk apparatus. The scores depended on the rats' behaviors, which allowed a score range of 0 to 10.
