# Supplementary material for: Tiagabine treatment in kainic acid induced cerebellar lesion of dystonia rat model
Source: EXCLI J. 2016 Nov 17;15:716–29. doi: 10.17179/excli2016-482 (PMC5318686; doi:10.17179/excli2016-482)
Supplement: Supplementary information [file EXCLI-15-716-s-001.pdf]

EXCLI Journal 2016;15:716-729 – ISSN 1611-2156
Received: June 28, 2016, accepted: October 28, 2016, published: November 17, 2016

Supplementary material to:

TIAGABINE TREATMENT IN KAINIC ACID INDUCED CEREBELLAR LESION OF DYSTONIA RAT MODEL

Tsui-chin Wang[1], Sukonthar Ngampramuan[2]*, Naiphinich Kotchabhakdi*

Research Center for Neuroscience, Institute of Molecular Biosciences, Mahidol University, Salaya campus, Nakhon Pathom 73170, Thailand

* Corresponding authors:
Sukonthar Ngampramuan: sukonthar.nga@mahidol.ac.th, Tel: +66-81-954-2959
Naiphinich Kotchabhakdi: naiphinich@gmail.com, Tel: +66-81-483-6066

http://dx.doi.org/10.17179/excli2016-482

This is an Open Access article distributed under the terms of the Creative Commons Attribution License (http://creativecommons.org/licenses/by/4.0/).

Abbreviations in the experiment: preS: pre-surgery, postS: post-surgery, sham: sham-operated group, KA: kainic acid/non-treated group, KA+TGB: kainic acid tiagabine treated group, TGB: tiagabine group, BW: beam walking, EMG: electromyography, DV: dorsal-ventral coordinates, i.p.: intraperitoneal injection.

EXCLI Journal 2016;15:716-729 – ISSN 1611-2156
Received: June 28, 2016, accepted: October 28, 2016, published: November 17, 2016

Supplemental information: raw data

S1. Dystonic behavioral scores for individual animal at 120 minutes post-surgery (Figure 3)

A

Gr./No.	Time (mins)											
**Sham**	10	20	30	40	50	60	70	80	90	100	110	120
1502	1	1	1	1	1	1	1	1	1	1	1	1
1504	0	1	1	1	1	1	1	0	0	0	0	0
1507	0	0	0	0	1	1	1	1	1	0	0	0
1512	2	2	2	1	2	2	2	1	1	1	1	1
Average	0.75	1	1	0.75	1.25	1.25	1.25	0.75	0.75	0.5	0.5	0.5
**KA**												
1402	6	6	6	5	5	6	6	6	6	4	4	3
1409	5	5	6	5	5	5	6	6	5	4	4	4
1410	5	5	4	6	6	6	6	6	6	6	6	6
1412	6	5	6	5	5	4	6	6	5	5	5	6
Average	5.5	5.25	5.5	5.25	5.25	5.25	6	6	5.5	4.75	4.75	4.75
**KA + TGB**												
1406	5	5	6	6	5	5	6	6	5	4	6	5
1408	5	4	4	6	4	6	5	5	5	6	5	6
1505	4	2	3	2	3	3	4	4	6	6	5	5
1508	4	4	5	4	3	5	6	6	5	4	5	6
Average	4.5	3.75	4.5	4.5	3.75	4.75	5.25	5.25	5.25	5	5.25	5.5

B

	Time (mins)											
Gr./No.	10	20	30	40	50	60	70	80	90	100	110	120
**Sham**	0.75	1	1	0.75	1.25	1.25	1.25	0.75	0.75	0.5	0.5	0.5
**KA**	5.5	5.25	5.5	5.25	5.25	5.25	6	6	5.5	4.75	4.75	4.75
**KA +TGB**	4.5	3.75	4.5	4.5	3.75	4.75	5.25	5.25	5.25	5	5.25	5.5

S1-Table 1: The dystonic scores from individual rats were obtained from dystonic behavioral scoring Table (Table 1 in the text). **(A)** Each group of rats was allotted a score every 10 minutes for 2 hours (10 to 120) in duration. The 4 digit numbers are the rat code numbers in each group. The average score for each group was calculated every 10 minutes after the scores were given, which has been highlighted with yellow for the sham-operated group, blue for the kainic acid group and pink for the kainic acid treatment group. **(B)** The different colors representing the average for each group was then used to plot a graph, Figure 3. Gr., group; No., rat code number; mins, minutes; Sham, sham-operated; KA, kainic acid; KA+TGB, kainic acid with tiagabine treatment.

EXCLI Journal 2016;15:716-729 – ISSN 1611-2156
Received: June 28, 2016, accepted: October 28, 2016, published: November 17, 2016

S2. Intra-muscular EMG amplitude data (Figure 4)

A

	No.	preS	postS	90m	7h	22h	30h	48h
**Sham**	1502	21.59	24.73	20.14	13.16	13.92	14.66	14.51
	1504	12.23	25.84	17.84	13.16	13.92	14.66	14.51
	1507	13.77	11.38	15.45	13.16	13.92	14.66	14.51
	1512	9.85	17.25	15.92	13.16	13.92	14.66	14.51
Average		14.364	19.804	17.341	13.164	13.92	14.668	14.519
SD		5.082	6.788	2.132	0	0	0	0

	No.	preS	postS	90m	7h	22h	30h	48h
**KA**	1402	14.88	29.93	28.03	30.44	40.73	49.94	47.54
	1409	13.14	36.11	39.72	27.62	38.97	43.75	46.98
	1410	11.44	48.57	12.80	34.37	49.87	48.63	55.78
	1412	19.66	54.16	29.30	47.78	49.72	57.89	40.36
Average		14.784	42.199	27.468	35.056	44.829	50.056	47.671
SD		3.545	11.122	11.091	8.924	5.785	5.862	6.315

	No.	preS	postS	90m	7h	22h	30h	48h
**KA+TGB**	1406	18.21	26.16	20.68	21.74	27.47	28.68	27.93
	1408	14.25	21.54	20.25	17.79	31.02	35.34	29.11
	1505	15.18	16.84	31.53	23.06	24.08	31.15	26.78
	1508	13.88	18.68	26.45	27.21	22.32	28.76	22.18
Average		15.385	20.809	24.732	22.456	26.229	30.897	26.501
SD		1.965	4.058	5.341	3.885	3.844	3.123	3.033

	No.	preS	postS	90m	7h	22h	30h	48h
**TGB**	1404	11.07	13.44	19.08	25.60	20.19	20.76	22.11
	1503	13.15	13.44	13.44	21.57	15.28	15.70	22.33
	1509	13.45	13.44	17.74	20.60	23.28	21.22	28.07
	1511	12.00	13.44	19.01	17.06	22.42	17.20	19.37
Average		12.424	13.442	17.320	20.213	20.295	18.724	22.975
SD		1.094	0	2.657	3.511	3.586	2.698	3.657

Received: June 28, 2016, accepted: October 28, 2016, published: November 17, 2016

B

Gr.	preS	postS	90m	7h	22h	30h	48h
**Sham**	14.364	19.804	17.341	13.164	13.92	14.668	14.519
SD	5.082	6.788	2.132	0	0	0	0
**KA**	14.784	42.199	27.468	35.056	44.829	50.056	47.671
SD	3.545	11.122	11.091	8.924	5.785	5.862	6.315
**KA+TGB**	15.385	20.809	24.732	22.456	26.229	30.897	26.501
SD	1.965	4.058	5.341	3.885	3.844	3.123	3.033
**TGB**	12.424	13.442	17.320	20.213	20.295	18.724	22.975
SD	1.094	0	2.657	3.511	3.586	2.698	3.657

S2-Table 1: The EMG data presented in this Table was summarized and averaged from the primary EMG amplitude in Chart 5.4. **(A)** The 4 digit numbers are the rat code numbers in each group. The amplitude of each duration, including the pre-surgery, post-surgery and hours after surgery were recorded from individual rats in each group. The average of each duration was calculated and highlighted in color; orange for the sham group, light green for the kainic acid group, pink for the kainic acid treatment group and blue for the tiagabine only group, with yellow as the standard deviation for each group. **(B)** The graph (Figure 4) was plotted using the average from each group. Gr., group; No., rat code number; Sham, sham-operated; KA, kainic acid; KA+TGB, kainic acid with tiagabine treatment; TGB, tiagabine only; preS, pre-surgery; postS, post-surgery; m, minutes; h, hour; SD, standard deviation.

EXCLI Journal 2016;15:716-729 – ISSN 1611-2156
Received: June 28, 2016, accepted: October 28, 2016, published: November 17, 2016

S3. Beam walking analysis

S3-1. Beam walking behavior observation: scores (Figure 5)

A.

Gr/ No.							
**Sham**	**preS**	**postS**					Average
1502	10	10	10	10	10	10	10
1504	10	10	10	10	10	10	10
1507	10	10	10	10	10	10	10
1512	10	10	10	10	10	10	10
							10
**KA**							
1402	10	0	0	0	0	0	0
1409	10	2	2	2	3	6	3
1410	10	2	2	3	3	6	3.2
1412	10	0	0	0	0	0	0
							1.57
**KA + TGB**							
1406	10	3	4	6	5	6	4.8
1408	10	5	4	4	4	6	4.6
1505	10	5	6	6	6	6	5.8
1508	10	6	6	8	8	8	7.2
							6
**TGB**							
1404	10	4	5	6	6	6	5.4
1503	10	10	9	10	8	9	9.2
1509	10	9	9	9	9	8	8.8
1511	10	9	10	10	10	10	9.8
							8

B.

	**preS**	**postS**
**Sham**	10	10
**KA**	10	1.57
**KA+TGB**	10	6
**TGB**	10	8

S3-Table 1: The behavioral scores were obtained in accordance with Table 2 from the text. **(A)** After being given the scores by a third person, before and after the surgery, the numbers were averaged from individual rats each group. **(B)** The average from each rat was calculated again and the final number (pink) from each group was used to plot a graph, Figure 5 from the text. The 4 digit numbers are the rat code numbers in each group. Gr., group; No., rat code number; Sham, sham-operated; KA, kainic acid; KA+TGB, kainic acid with tiagabine treatment; TGB, tiagabine only; preS, pre-surgery; postS, post-surgery.

EXCLI Journal 2016;15:716-729 – ISSN 1611-2156
Received: June 28, 2016, accepted: October 28, 2016, published: November 17, 2016

S3-2. Pre-surgery (training/habituation) and post-surgery: average time in crossing the bridge for individual rat. Time in seconds (Figure 6A)

A1.

Gr./No.	Pre-surgery										
**Sham**	Session 1					Session 2					Average
1502	11.97	13.54	11.86	9.18	12.59	9	13.68	14.7	16.06	16.01	12.85
1504	8.61	7.07	9.59	6.86	6.92	12.71	8.37	13.42	6.26	17.35	9.716
1507	7.65	5.32	14.36	13.53	15.41	16.71	9.01	6.42	5.45	9.14	10.3
1512	13.26	9.51	10.21	11.42	10.74	14.75	12.25	9.04	7	10.91	10.90
											10.95
**KA**											
1402	3.85	6.67	4.36	10.96	12.54	7.44	5.57	12.31	7.96	10.15	8.18
1409	11.38	9.69	17.64	16.17	8.4	5.37	16.14	3.73	3.78	4.82	9.71
1410	19.62	12.18	9.24	18	14.42	9.58	5.59	6.78	4.25	9.76	10.94
1412	7.93	5.3	6.12	8.7	10.9	7.6	8.75	6.88	12.76	18.13	9.30
											9.53
**KA + TGB**											
1406	15.57	9.27	12.22	10.05	10.23	8.21	13.71	11.79	7.24	14.04	11.23
1408	5.54	8.75	8.87	6.66	6.22	10.29	5.39	6.53	11.21	13.22	8.26
1505	9.74	8.04	7.78	9.45	12.4	7.95	10.22	17.31	11.98	13.27	10.81
1508	12.16	15	12.12	6.14	9.37	15.13	13.12	12.95	10.45	19	12.54
											10.71
**TGB**											
1404	16.15	13.17	12.45	12.16	9.97	8.72	9.72	5.73	7.89	6.29	10.22
1503	6.86	6.45	5.78	8.87	7.33	10.04	11.12	6.45	11.13	5.3	7.93
1509	8.87	9.35	10.1	8.87	9.01	6.19	8.61	9.39	11.33	11.67	9.33
1511	9.89	8.36	9.98	9.88	9.53	8.8	13.96	11.47	7.73	7.39	9.69
											9.29

EXCLI Journal 2016;15:716-729 – ISSN 1611-2156
Received: June 28, 2016, accepted: October 28, 2016, published: November 17, 2016

A2.

Gr./ No.	Post-surgery			
**Sham**				Average
1502	14.13	10.12	14.48	12.91
1504	8.02	18.62	12.29	12.97
1507	9.55	17.45	15.67	14.22
1512	13.34	11.22	10.91	11.82
				12.98
**KA**				
1402	60	60	60	60
1409	7.15	16.01	50.96	24.7
1410	20.71	60	22.65	34.45
1412	60	60	60	60
				44.78
**KA + TGB**				
1406	14.88	21.29	60	32.05
1408	12.54	30.53	20.2	21.09
1505	60	18.79	22.48	33.75
1508	11.93	14.62	16.64	14.39
				25.32
**TGB**				
1404	21.2	10	21.12	17.44
1503	15.55	15.78	13.47	14.93
1509	16.16	20.2	33.02	23.12
1511	14.66	9.56	14.35	12.85
				17.08

B.

	**preS**	**postS**
**Sham**	10.95	12.98
**KA**	9.53	44.78
**KA+TGB**	10.71	25.32
**TGB**	9.29	17.08

S3-Table 2: As mentioned in the main text, individual rat was trained for two sessions before the surgery. **(A1)** The pre-surgery data was obtained during the two sessions and averaged from individual rats in each group and highlighted according to the group; yellow for the sham group, orange for the kainic acid group, green for the kainic acid treatment group and blue for the tiagabine only group, with further averaging highlighted in pink. **(A2)** The record for the 3rd beam walking task was also calculated from individual rats after the surgery, with the average highlighted in light green for the sham group, grey for the kainic acid group, green for the kainic acid treatment group and purple for the tiagabine group. **(B)** Pre-surgery and post-surgery data were compared and plotted into a graph as Figure 6A. The 4 digit numbers are the rat code numbers in each group. Gr., group; No., rat code number; Sham, sham-operated; KA, kainic acid; KA+TGB, kainic acid with tiagabine treatment; TGB, tiagabine only; preS, pre-surgery; postS, post-surgery.

EXCLI Journal 2016;15:716-729 – ISSN 1611-2156
Received: June 28, 2016, accepted: October 28, 2016, published: November 17, 2016

S3-3. Post-surgery: distance crossed (cm) (Figure 6B)

Gr/ No.				
**Sham**	**postS**			Average
1502	110	110	110	110
1504	110	110	110	110
1507	110	110	110	110
1512	110	110	110	110
				110
**KA**				
1402	0	0	0	0
1409	30	110	110	83.33
1410	80	0	110	63.33
1412	0	0	0	0
				36.5
**KA + TGB**				
1406	60	60	0	40
1408	50	110	110	90
1505	0	90	40	43.33
1508	110	110	110	110
				70
**TGB**				
1404	70	70	70	70
1503	110	110	110	110
1509	110	110	110	110
1511	110	110	110	110
				100

S3-Table 3: The distance in crossing the beam was measured in the beam walking task after the surgery. Data from each rat was calculated (blue) and the final number for each group (green) was used to plot a graph, as Figure 6B in the text. The total length of the beam was 110 cm. The 4 digit numbers are the rat code numbers in each group. Gr., group; No., rat code number; Sham, sham-operated; KA, kainic acid; KA+TGB, kainic acid with tiagabine treatment; TGB, tiagabine only; postS, post-surgery.
